# Advancing Medical Education in a Mental Health Trust: Trainees’ and Students’ Perspectives – Focus Groups

**DOI:** 10.1192/bjo.2025.10383

**Published:** 2025-06-20

**Authors:** Michelle Keag, Varoonan Sritharan, Bruce Tamilson

**Affiliations:** 1 South West London and St George’s NHS Mental Health Trust, London, United Kingdom; 2 St George’s University of London, London, United Kingdom.; 3 St George’s Hospital, London, United Kingdom.; 4 Kingston Hospital, London, United Kingdom

## Abstract

**Aims:** Medical education is a cornerstone of the NHS, influencing the continuous improvement of patient care. This study aims to explore the experiences of psychiatric trainees and medical students within a mental health trust, identifying opportunities to enhance medical education quality.

**Methods:** A qualitative focus group methodology was employed to capture detailed perspectives of participants. Four separate focus groups were conducted, categorized by the level of training: medical students (n=4), foundation doctors (n=4), core psychiatric trainees (n=8), and higher specialty trainees (n=4). Participants were recruited via internal mass email. Focus groups were guided by a standardized topic guide. Data were analysed using thematic analysis.

**Results:** Key findings reveal shared themes of issues in induction processes, access to information, rota issues, and facilities. Additionally, the study highlighted the importance of structured support for achieving psychotherapy competencies and the necessity for both clinical and non-clinical training. There is also a significant need for better supervision, support, and appreciation.

**Conclusion:** The study provides insights into the experiences of psychiatric trainees and medical students, highlighting key areas for improvement. Implementing the practical recommendations can enhance medical education quality within mental health trusts, benefiting trainees, educators, and patients.

